# “When I Think of Black Girls, I Think of Opportunities”: Black Girls' Identity Development and the Protective Role of Parental Socialization in Educational Settings

**DOI:** 10.3389/fpsyg.2022.933476

**Published:** 2022-07-25

**Authors:** Marketa Burnett, Margarett McBride, McKenzie N. Green, Shauna M. Cooper

**Affiliations:** ^1^Department of Psychology, University of South Carolina, Columbia, SC, United States; ^2^Department of Psychology and Neuroscience, University of North Carolina at Chapel Hill, Chapel Hill, NC, United States; ^3^Department of Psychology, Virginia Commonwealth University, Richmond, VA, United States

**Keywords:** Black girls, parental socialization, schools, identity, adolescent development

## Abstract

While educational settings may be envisioned as safe spaces that facilitate learning, foster creativity, and promote healthy development for youth, research has found that this is not always true for Black girls. Their negative experiences within educational settings are both gendered and racialized, often communicating broader societal perceptions of Black girls that ultimately shape their identity development. Utilizing semi-structured interviews with adolescent Black girls (*n* = 12), the current investigation explored Black girls' educational experiences, their meaning making of Black girlhood, and the role of parents in their positive development. By centering Black girls' voices, this study illuminated how Black girls negotiate their multiple marginalized identities and how their identities are shaped by their home and school environments. Findings revealed that Black girls are aware of the difficulties in navigating educational settings for Black girls, but this awareness was coupled with parental support that promoted positive gendered racial identities for Black girls in middle school and high school. This investigation advanced current knowledge of Black girls' identity development and highlighted the protective role of parental socialization. Future research directions and implications are also discussed.

## Introduction

“When I think of Black girls, I think of opportunities. And I think of options. And I think of possibilities. But I also think of harder workers and having more to do and not a fair playing field.”

    Ruth, 8th grade, 13 years old

Empirical and theoretical research highlights that Black girls' experiences within educational settings are both gendered and racialized, including interactions with peers, teachers, as well as broader systemic school policies (Chavous and Cogburn, 2007; Evans-Winters and Esposito, 2010; Evans-Winters, 2014; Morris and Perry, 2017; Neal-Jackson, 2018). For example, compared to students of other races, research has found that teachers and counselors report lower educational expectations and perceptions of academic engagement and motivation for Black girls (West-Olatunji et al., 2010; Archer-Banks and Behar-Horenstein, 2012; Pringle et al., 2012; Neal-Jackson, 2018; Carter Andrews et al., 2019). Further, Pringle et al. (2012) found that teachers expressed lower educational expectations of Black girls, especially when positioned as math and science learners. Instead, research conducted by Francis (2012) indicated that teachers were more likely to perceive middle school aged Black girls as more disruptive than their White, Asian, and Hispanic peers even after controlling for academic performance and socioeconomic status. According to Epstein and colleagues (2020) report, Black girls are 4.19 times more likely to be suspended and 3.99 times more likely to be expelled compared to White girls. Studies have linked the prevalence and subsequent implications of teacher-based discrimination and disproportionate discipline policies on Black girls' academic and psychosocial adjustment (Blake et al., 2011; Bryan et al., 2018; Leath et al., 2019; Butler-Barnes and Inniss-Thompson, 2020; Hines-Datiri and Carter Andrews, 2020; Cooper et al., 2022).

While educational spaces may be envisioned as safe spaces for many youth, studies specific to Black girls have found that Black girls experience discrimination in their school and classroom contexts (Morris, 2007; Morris and Perry, 2017; Nunn, 2018; Carter Andrews et al., 2019). These experiences communicate broader societal perceptions of Black girls' academic abilities and inform their identity development. Using a qualitative framework, Mims and Williams (2020) found that Black girls reported experiencing bullying by their peers that often invoke stereotypical language and imagery learned within the classroom (i.e., being called slaves during history lessons). Archer-Banks and Behar-Horenstein (2012) suggest that Black girls' academic identity is molded by their experiences in the classroom and broader school context, underscoring how school contexts may be “racially stigmatizing.” As Black girls begin to recognize the ways their lives are dually gendered and racialized, they often seek the guidance of additional socialization agents, such as their parents, to process these experiences and seek adaptive ways to cope and navigate school settings. This is particularly true during adolescence, which is a developmental period often marked by increasing awareness of bias and experiences of racism and discrimination (Seaton and Tyson, 2019; English et al., 2020; Tynes et al., 2020). Umaña-Taylor et al. (2014) explain that this greater awareness emerges as result of increased social-cognitive maturity which can (1) lead adolescents to merge their personal sense of self with their racial group and (2) explore different meanings of race beyond what their parents tell them. Thus, racial socialization remains valuable during adolescence as Black girls experience a new racial reality, but it is also complicated by normative shifts in identity development when youth become more autonomous and potentially less receptive to their parents' socialization efforts (Smetana et al., 2015).

Nonetheless, parents cannot shield their Black daughters from the realities of individual and structural level discrimination. Studies have, however, highlighted the ways Black mothers and fathers have supported their children in ways that are promotive of healthy identities and protect against the negative effects of discrimination (Murry et al., 2018; Anderson and Stevenson, 2019; Cooper et al., 2020; Umaña-Taylor and Hill, 2020; Jones et al., 2021). Noting the historical context of the Black experience in the United States, many Black parents engage in conversations with their children that reflect race-related concerns and expectations, which is defined as parental ethnic-racial socialization (Hughes et al., 2006; Anderson and Stevenson, 2019; Cooper et al., 2020; Jones et al., 2021). There is a robust body of research that positions parental ethnic-racial socialization as beneficial for Black youth. The current study expands this work by considering concurrently communicated dimensions of ethnic-racial socialization at the intersections of gender and academic socialization (Cooper and Smalls, 2010; Stokes et al., 2020; Cunningham, 2021; Huguley et al., 2021).

Seeking to provide a comprehensive approach and further appraise the study of positive development among Black girls, this investigation centers Black girls' voices as they articulate their own educational experiences and how they have informed their understanding of Black girlhood. As part of this broader landscape, we also contend that Black parents utilize a variety of messages and strategies to actively resist the negative portrayals of Black girls (e.g., disengaged; disruptive) and combat inequitable practices in K-12 school settings. Leveraging qualitative methods, the current study expands our understanding of the tools and strategies Black parents equip their daughters with to facilitate healthy identity development while also promoting academic engagement. Together, this study demonstrates the collective influence of home and school contexts in the development of Black girls.

## Literature Review

### Ethnic-Racial Socialization

Ethnic-racial socialization (ERS) encompasses the messages, behaviors, and strategies parents share with children regarding race and culture (Hughes et al., 2006; Brown et al., 2010). Scholars have highlighted the multidimensional nature of ERS (Hughes et al., 2006) and the literature emphasizes key dimensions such as egalitarian (strategies that emphasize the individual over groups due to the belief that all groups are equal, often paired with color-evasive perspectives), racial pride (the teaching of cultural knowledge, values, customs, and traditions), preparation for bias (preparing youth for racism/discrimination and providing strategies to cope) and promotion of mistrust (Hughes et al., 2006; Umaña-Taylor and Hill, 2020; messages that emphasize distrusting other ethnic-racial groups). Research has found that ERS messages are often promotive and protective and lead to a positive impact on a variety of psychosocial and academic outcomes in Black youth (Neblett et al., 2006; Wang and Huguley, 2012; Varner et al., 2018; Anderson and Stevenson, 2019; Umaña-Taylor and Hill, 2020). For example, Wang and Huguley (2012) found that cultural socialization messages (positive messages about one's racial group related to pride, history and tradition) attenuated the negative effect of teacher and peer discrimination on Black youth's educational aspirations and GPA. Banerjee et al. (2018) found that when adolescents reported high levels of preparation for bias and cultural socialization messages from their parents, ERS acted as a buffer against the effects of peer discrimination on Black youth's academic persistence, self-efficacy, and self-concept. However, when low levels of ERS messages were reported, peer discrimination was associated with less favorable outcomes (Banerjee et al., 2018). It is important to note that additional studies have found no significant relationship or, in some cases, a negative relationship, between ERS messages and both psychosocial and academic outcomes depending on the ERS dimension (McHale et al., 2006; Neblett et al., 2006; Smalls, 2009; Umaña-Taylor and Hill, 2020). The equivocal nature of this literature has pointed to the importance of considering the frequency and intensity of ERS messages as well as the broader relational context between a parent and child (Coard et al., 2004; Cooper and McLoyd, 2011; Umaña-Taylor and Hill, 2020).

Research has also suggested that parents' ERS messages and motivations for employing these messages may differ based upon parent and child gender (Bowman and Howard, 1985; Thomas and Speight, 1999; Brown et al., 2010; Caughy et al., 2011; Cooper et al., 2020). For instance, Brown et al. (2010) found that, though both mothers and fathers engaged in ERS messaging, adolescents reported that maternal caregivers engaged in more ERS messaging across dimensions. Additionally, compared to the adolescent boys in this sample, the adolescent girls reported receiving more ERS messaging from their parents (Brown et al., 2010). Bowman and Howard (1985) found that girls received more racial pride messaging, whereas boys reported more egalitarian and racial barrier (preparation for bias) messages from their parents. Studies which highlight Black parents' differential motivations and messaging for their sons and daughters, provides some support for gendered ERS (Bowman and Howard, 1985; Thomas and Speight, 1999; Hughes et al., 2006; Cooper et al., 2020). For instance, Cooper et al. (2020) qualitative investigation found that Black fathers had distinct gendered ERS motivations, including catalysts and strategies for supporting their daughters' and sons' development. For example, fathers in this sample emphasized messages around education and positive self-image with their daughters compared to messages about personal safety with their sons. Collectively, these studies suggest the need to capture motivations and catalysts for ethnic-racial socialization.

### Gendered Racial Socialization

Compared to their sons, research indicates that Black parents communicate distinct types of ERS messages to their daughters. Thomas and Speight (1999) work helped to ground later exploration into gendered racial socialization of Black girls as they found that Black girls received messaging focusing on education, beauty, sex, relationships, racial pride, and independence compared to boys' messaging regarding the awareness of and overcoming racial barriers and egalitarianism. Building on these findings, Thomas and King (2007) employed a mixed-method design to understand gendered racial socialization among Black mother-daughter dyads. Findings revealed that coded themes were similar between mothers and daughters, including a joint emphasis on messages around self-determination, self-pride, racial pride, and spirituality. Overall, themes from this investigation suggested that, as a *double minority*, Black girls share a unique developmental context and socialization may help them navigate and cope with gendered racism (Lewis et al., 2013). Parents then often engage in gendered racial socialization, sharing messages with their daughters that prepare them for these realities and provide strategies to ensure that their daughters are still able to develop a healthy and positive self-image in the face of gendered racism.

Using a sample of college-aged Black women, Brown et al. (2017) developed a gendered racial socialization measure (Gendered Racial-Ethnic Socialization Scale for Black Women; GRESS-BW) to retroactively capture the frequency of gendered racial socialization messages women received from their parents. The exploratory factor analysis revealed nine factors across 63 items: gendered racial pride and empowerment; family expectations and responsibilities; internalized gendered racial oppression; independence, career, and educational success; sexual behavior; oppression awareness; sisterhood; religious faith and spirituality; gendered racial hardship. Additional validation will be required, but the initial development of the GRESS-BW is a promising advancement in understanding the messages that Black girls receive from their parents that will ultimately shape their identity. Though little work has yet to use this scale with adolescent girls, Stokes et al. (2020) recent investigation, the first of its kind to use (GRESS-BW) with adolescents, confirms that subscales of GRESS-BW are reliable among a sample of adolescent Black girls. Girls reported receiving a high frequency of gendered racial pride and empowerment messages and far fewer messages regarding internalized gendered racial oppression. Both tested dimensions of gendered racial socialization were positively associated with girls' racial identity. This is particularly important to note as racial identity was indirectly and directly associated with Black girls' depressive symptoms. This novel study underscores the necessity of considering how gendered racial socialization may shape Black girls' identity and reflect their lived experiences.

### Academic Socialization

One common theme in the gendered racial socialization literature is the emphasis that Black parents of girls place on the importance of academic achievement for advancement (Thomas and Speight, 1999; Brown et al., 2017; Cooper et al., 2020). Collectively, these studies suggest the need to explore the content and frequency of parents' discussion of academics with their Black daughters through academic socialization. Academic socialization can be defined as the messages, behaviors, and expectations that parents share with their children to encourage and foster academic development and adjustment (Taylor et al., 2004). Black parents' school involvement extends beyond the classroom, as they often participate in a variety of activities that promote academic interest and engagement with their children (Martin, 2006; Cooper and Smalls, 2010; Archer-Banks and Behar-Horenstein, 2012; Latunde and Clark-Louque, 2016). For instance, Latunde and Clark-Louque (2016) found that 60% of Black parents engaged in educational activities, such as visiting museums and attending weekly educational-based camps. Utilizing individual interview and focus group data, Archer-Banks and Behar-Horenstein (2012) revealed that Black girls received various academic socialization messages from their parents, ranging from monitoring academic progress and homework completion to encouraging enrollment in advanced rigorous courses. Additionally, girls mentioned the roles of parents and grandparents as a place of support as they encouraged them to persevere despite obstacles that they were facing within school contexts (Archer-Banks and Behar-Horenstein, 2012).

Huguley et al. (2021) contend that previous research on parental involvement (home and school-based) and academic socialization centers the lived experiences of White and middle-class families, ignoring critical cultural and developmental contexts and misses the ways Black parents facilitate the educational advancement of their children through culturally relevant strategies such as how to cope with school-based discrimination (Huguley et al., 2021). Themes derived from focus groups and interviews with Black parents suggested two domains of racialized academic socialization: cultural academic socialization and racial bias academic socialization. *Cultural academic socialization* was defined as “socializing activities at home that were designed to promote positive racial identities and academic motivation simultaneously.” Racial bias academic socialization incorporated the ways parents helped their children cope with implicit and overt discrimination experiences that could impede their academic progress and success (Huguley et al., 2021). Similar to the benefits of ERS, previous research has found that academic socialization is linked to positive academic outcomes among Black youth (Cooper and Smalls, 2010; Metzger et al., 2020). For example, Cooper and Smalls (2010) found that increased academic involvement and educational encouragement (academic socialization) from parents were associated with greater academic engagement and higher academic self-esteem in Black middle school-aged youth. Recent work by Metzger et al. (2020) further explores how socialization practices influence Black youths' academic development and adjustment by identifying various socialization profiles at the intersection of race and academic socialization. The most common profile of the sample was of parents who were multifaceted socializers. These parents reported moderate academic involvement, educational encouragement, racial pride, preparation for bias, and egalitarian messages. Results found that adolescents whose parents were classified as multifaceted socializers reported significantly greater academic self-beliefs than those whose parents were in the preparation for bias socializers group. These findings further confirm the need for future studies exploring academic socialization through a culturally situated lens.

### Theoretical Foundations and Goals of the Study

Acknowledging the need for a model that considers how Black children process and make meaning of their lived experiences, Spencer proposed a model called the Phenomenological Variant of Ecological Systems Theory (PVEST). Within the Spencer (1995) model, PVEST begins with acknowledging the effects of self-other appraisals—how one perceives how others view them with special consideration to both biases and stereotypes in a host of domains (e.g., race, gender, and socioeconomic status). PVEST posits that how someone makes meaning of these self-other appraisals also impacts their stress engagement which leads to their reactive coping strategies. It is through these reactive coping strategies—adaptive and/or maladaptive, through which stable identities begin to emerge. Among the multiple identities that can emerge, Spencer (1995) highlights how people identify who they are regarding their culture and ethnicity, gender role identities, and even their own self-efficacy. Lastly, these emerged identities consequently lead to select health and behavioral related outcomes. In this model, Spencer (1995) emphasizes that these outcomes can both be adverse (e.g., deviance, poor health) or productive (e.g., competence, healthy relationships). The emphasis on meaning-making within PVEST is relevant to this study as we seek to understand *how* Black girls' identity development is shaped by the home and academic context. This investigation is especially interested in exploring the adaptive strategies Black parents employ to help their daughters navigate their educational experiences and maintain a healthy sense of self. Further evaluation into Black girls' negotiation of their multiple identities in relation to their educational experiences is needed.

Building upon this theoretical foundation, the proposed study employs a theoretical thematic analysis (Braun and Clarke, 2006) to understand how various forms of parental socialization may contextualize and influence Black adolescent girls' identity development. Our use of this approach is strengthened by PVEST, which emphasizes youths' own meaning-making processes and recognizes their critical role as interpreters of their own experiences. The proposed research has two main research questions: (1) How are Black girls making meaning of Black girlhood and in what ways might their identity processing be related to parental socialization? and (2) Are there differences in the identity processing and the content of reported parental socialization of Black girls in middle school compared to Black girls in high school?

## Methods

### Participants

Thirteen adolescents who self-identified as Black girls agreed to participate in the current investigation. One participant chose to end the interview during the first question and has been excluded from the analysis, resulting in a final sample of 12 adolescent Black girls (Mage = 13.66). Recent studies focused on Black girls' identity development and school experiences have been published with samples of a comparable size (Mims and Williams, 2020; Mayes et al., 2021; Rogers and Butler-Barnes, 2022), which further illustrates that this sample size (*n* = 12) is effective for examining our research aims. This investigation had an even split in which six of the participants were currently in middle school and six participants were currently in high school. Seven girls resided in or attended school in City 1 while five girls resided in or attended school in City 2. In the current sample, 11 of the participants attended a public school and one participant attended a private religious-based school. For girls in this sample, the percentage of Black students at their schools ranged from 3.4 to 78.2% (NCES, 2022). Additionally, the percentage of students in their school that were eligible to receive free or reduced lunch ranged from 19.7 to 99.8% (NCES, 2022). All participants were assigned a pseudonym to protect their confidentiality. Full participant demographics can be found in Table 1.

**Table 1:** Participant demographics.

**Pseudonym**	**Age**	**City**	**Grade level**	**School racial demographics**	**Free/reduced lunch**
Dorothy	16	City 2	10th	36% Black	47.9%
Marie	12	City 2	7th	41.6% Black	51.2%
Alexa	11	City 1	6th	46.0.% Black	53.0%
Shirley	11	City 1	6th	41.3% Black	51.4%
Margaret	14	City 2	9th	37.8% Black	35.9%
Mary	12	City 1	7th	48.2% Black	30.8%
Katherine	16	City 2	11th	43.4% Black	38.9%
Marsha	17	City 1	12th	3.4% Black	N/A
Patricia	13	City 1	8th	48.2% Black	30.8%
Ruth	13	City 1	8th	48.2% Black	30.8%
Gladys	15	City 1	9th	27.9% Black	19.7%
Mae	14	City 2	9th	78.2% Black	99.8%

### Procedure

After receiving approval from the [University of North Carolina at Chapel Hill Institution Review Board] Institution Review Board, Black adolescent girls were recruited for individual interviews. The current study employed targeted online efforts to reach Black parents of middle and high school girls living in or attending school in two cities in the Southeastern region of the United States. Also, participants were given a flier at time of recruitment to share with anyone in their network that was also eligible to participate in the study. Due to similar demographic make-ups and vast school choice alternatives, the two cities were intentionally selected for targeted recruitment. Both cities were racially diverse with median household incomes of $52,106 and $45,787 in city 1 and city 2, respectively. Though variation within school resources, course offerings, and teacher qualifications exists, the opportunity for students to attend magnet schools outside of their residential zone was available in both counties.

In the current investigation, 12 semi-structured interviews were conducted with adolescent Black girls. Interviews had an average duration of 32 min. However, there was variability in interview duration, with interviews lasting between 23 and 42 min. Before beginning the interview, participants were reminded of the study's goals, the researcher's responsibility in protecting their privacy and confidentiality, and potential risks and benefits of the study. Participants also were told that they could refuse to answer any questions or conclude participation in the interview at any time. All interviews began with an initial rapport building discussion. The developed interview protocol used a variety of prompts to discuss three topics: (1) understanding of and meaning making around Black girlhood, (2) general attitudes and experiences regarding school and STEM, and (3) messaging received from parental figures. A full list of the structured questions asked during the interview can be found in the Supplementary Materials.

### Positionality Statements

The lead author is a Black woman who grew up in the South. Though my parents did not have many explicit conversations about what it means to be Black, I was always surrounded by Black families, culture, and traditions. I grew up in the Black church and was born in a city rich with a history of activism. I was always proud to be Black. I grew up in the free and reduced-price lunch program and attended public schools that were lower resourced. Through my parents' advocacy, I was eventually placed on an academic track that granted me access to honors and advanced courses throughout my K-12 education. My parents always made sure that I knew that I was smart and capable, immersing me in messages around the importance of education. They maintained high academic expectations. College was never a choice. My research is grounded in resilience frameworks (Spencer, 1995; Garcia Coll et al., 1996) that acknowledge cultural assets and multifaceted supports that facilitate Black girls' positive development. Through my work, I strive to honor and accurately represent Black girls' emerging identities and lived experiences as they navigate their social and academic contexts.

The second author is a Black woman who was raised in the North. I grew up in an under-resourced, Black neighborhood and had a racially and economically-diverse extended family, which made my understanding of race and class more apparent from a young age. My awareness of my race, class, size, and gender led to many conversations from my parents about how to navigate our neighborhood, city, country, and world. My parents not only made sure that I was aware of why it was beautiful to be Black, but also discussed the historical and contemporary barriers that Black people have to face. They value education, community, and integrity. I have experience in my mostly Black neighborhood elementary school (Pre-K; 2–5th grade), a mostly White gifted/talented elementary school briefly (K, 1st grade), and a racially diverse gifted/talented program for middle and high school. For my secondary education, I attended Predominately White Institutions and led a Black women focused organization on campus. My work involves telling authentic and dynamic stories about Black families and neighborhoods in a way that acknowledges the adversity and uplifts the processes and factors that keep us here today.

The third author is a Multiracial-Black woman who was raised in the Western region of the United States by her single white mother. The area that I lived in was extremely rural and over 90% of the residents were white, so I was often one of the only Black children in the neighborhoods and schools that I grew up in. I was frequently stereotyped and discriminated against within these spaces based on the intersections of my race, gender, and class which resulted in me experiencing harsher disciplinary infractions and fewer academic opportunities like taking advanced placement courses than my peers. My mother did attempt to implicitly advocate on my behalf within educational spaces, but would rarely discuss matters of racism or race generally with me. My father, who is Black, lived in the Northeastern region of the U.S., so he was often unable to intervene but he did his best to expose me to positive messaging about Black people and culture to make sure that I was proud to be Black. We also talked frequently about the unique barriers, like gendered racism, that I was facing as a Black girl in my rural hometown and how I could cope with it through activism and resistance. My research now considers how these intersectional conversations about race and gender can shape the identity and wellbeing of Black and Multiracial-Black youth during critical periods of development.

The fourth author is a Black woman who was raised in the Southeastern United States. My mother is Black American. My father is Caribbean and immigrated to the United States as a young adult. I grew up in a small, agricultural town, located outside of a metro area. Several generations of my family also lived in close proximity to this area, providing connections to my large extended family and access to community and fictive kin supports. My parents, though divorced, emphasized the importance of Black cultural heritage as well as historical and present racial barriers impacting the Black community. My parents and extended family endorsed the value of education, the power of voice, and community preservation. Also, my awareness of race, equity, class, and gender was catalyzed by my own interactions and observations as a Black female residing in a predominately White community, attending overwhelmingly White schools in my K-12 education. My post-secondary educational institutions were Predominately White Institutions (PWI), though I was a member of multiple cultural and social justice organizations. Currently, as a tenured faculty at a PWI, my work has been grounded in strengths-based approaches that disrupt deficit narratives about Black families and communities, while also understanding how racism and structural inequities can subvert wellbeing across the life span.

## Data Analysis

### Transcription of Interviews

All video interviews were recorded, and audio files were then downloaded for transcription. Transcription was done verbatim by using the automated transcription capabilities found in Zoom. All identifying information (e.g., child's name, school, and teacher's name) was redacted from the transcripts to protect the privacy and confidentiality of each participant. Transcripts were then reviewed to correct any inaccuracies and improve the quality of the transcript in the case of inaudible instances by three self-identifying Black undergraduate research team members (one cisgender man, one cisgender woman, and one non-binary individual) and the principal investigator (Black cisgender woman).

### Codebook Development and Refinement

Codes were developed in a way that closely aligned with PVEST and our research questions (Braun and Clarke, 2006). PVEST (Spencer, 1995; Spencer et al., 1997), as our guiding theoretical foundation, emphasizes youths' meaning-making about multiple aspects of their identity formation, while also considering the varied coping strategies they may employ in response to their lived experiences. As demonstrated in other investigations (Spates et al., 2020), theoretical thematic analysis provided the opportunity for study researchers to explore Black girls' processing around what it means to be a Black girl and how girls perceived their social positioning within their educational settings. Also, as the broader project sought to explore the role of parental socialization on Black girls' identity development and STEM engagement, the development of codes and the codebook were guided by existing frameworks and conceptualizations of gendered-racial identity, racial identity, ethnic-racial socialization, gendered racial socialization, academic socialization, and STEM socialization among Black families (Sellers et al., 1997; Hughes et al., 2006; Brown et al., 2017; Huguley et al., 2021; Williams and Lewis, 2021).

**Figure 1:**
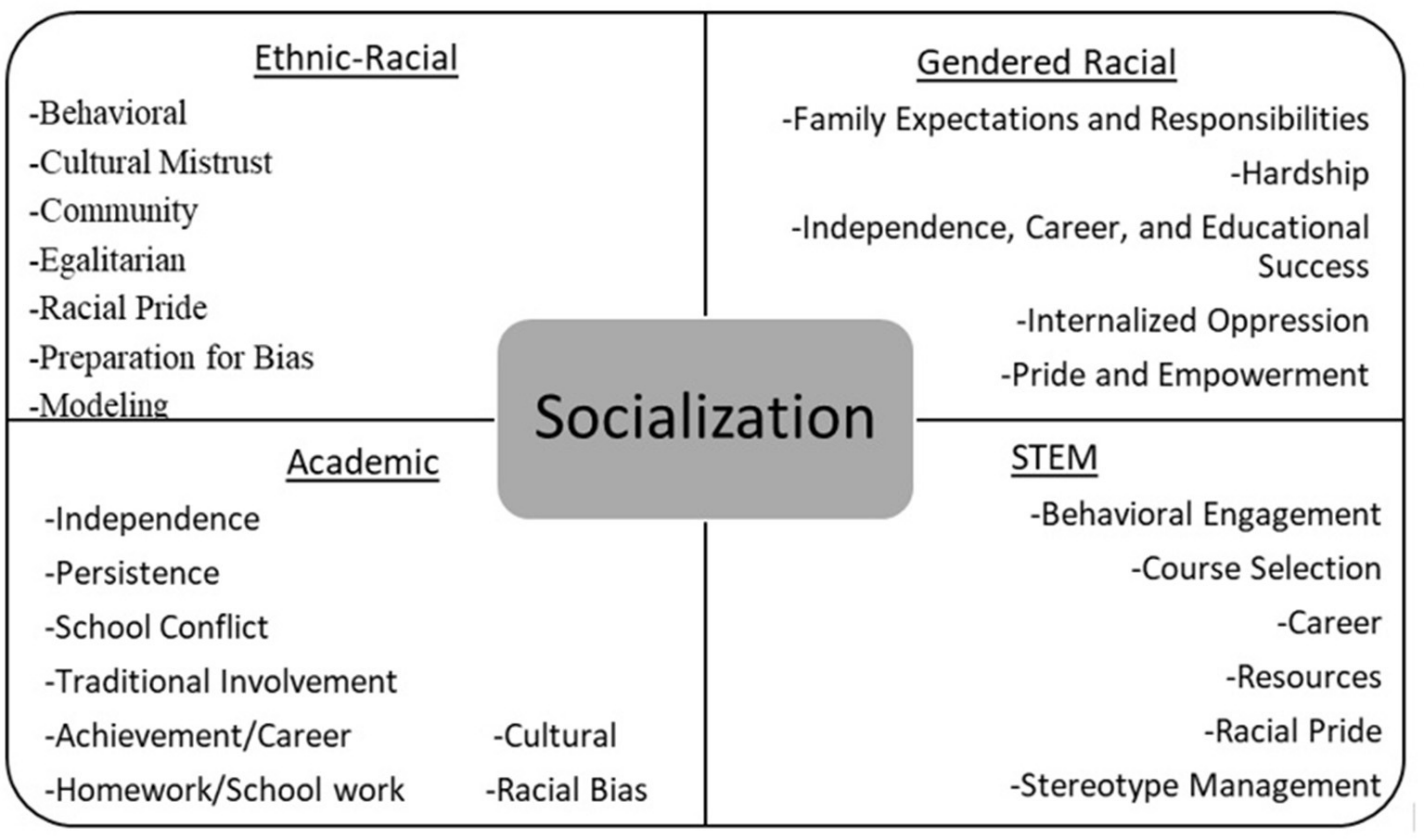
Socialization codes and subcodes.

**Figure 2:**
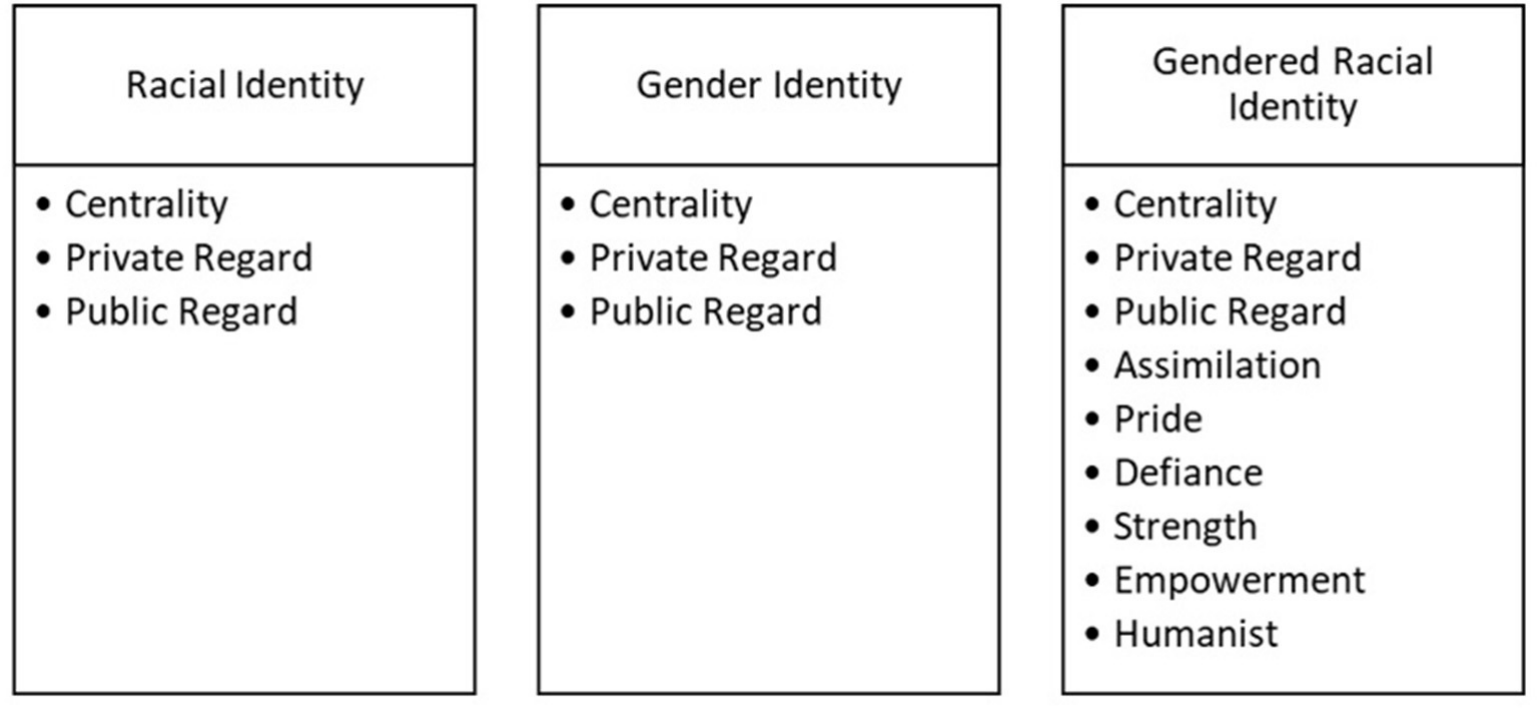
Identity codes and subcodes.

Building upon a qualitative protocol outlined in Cooper et al. (2020), an initial codebook was developed by the principal investigator to encompass seven overarching codes across identity and socialization: gender identity, racial identity, gendered racial identity, ethnic-racial socialization, gendered racial socialization, academic socialization, and STEM socialization. Within each of the seven general codes, subcodes were created to provide greater specificity into the ways these constructs emerged in the interviews. Subcodes were chosen based on previous theoretical and empirical literature regarding identity development and parental socialization dimensions among Black girls and Black youth, more broadly. A second member of the research team, a Black woman who is an advanced graduate student with expertise in Black family processes, reviewed the codebook, asked clarifying questions, and proposed additional subcodes to be included in the codebook. After discussion, researchers revised the codebook and agreed to continue refining the codebook as necessary throughout the coding process. The completed codebook included descriptions and example codes to ensure that coding remained consistent across participants. Below you will find a visual depiction of the main codes and subcodes related to socialization (see Figure 1) and identity (see Figure 2).

### Coding Approach

After all interview transcripts had been deidentified and reviewed for accuracy, they were uploaded into the Dedoose (Version 9.0.46) cloud-based data software program for coding. To ensure that the selected data codes were the best representation of the participants' voices, coding took place in multiple iterations. The lead investigator and an advanced doctoral level student completed coding. Researchers coded the full interview transcript to ensure all instances of identity development and socialization were included in analysis. As messaging around identity development and socialization practices are often happening in tandem, researchers employed the use of simultaneous coding (Saldaña, 2021).

Disagreements in the definition and application of codes were discussed and reconciled throughout the coding process to improve the quality and consistency of future coding iterations. Coders were intentional in discussing how their lived experiences as Black women who were once Black girls as well as their own biases may shape their initial interpretations of the data. To best represent the participants' lived experiences, researchers assigned codes based on the verbatim language used by participants, always situated within the context that participants provided. To determine intercoder reliability, percent agreement between coders for all transcripts was calculated. For each coded transcript, the number of agreements were divided by the number of total codes accumulated between the two coders initially (agreements/ agreements + disagreements) and then multiplied by 100 to acquire a percentage (Miles and Huberman, 1994). For the current investigation, we had an initial percent agreement of 74.48% between coders. Guidelines for adequate intercoder reliability estimates are mixed, however experts often recommend a coding agreement of at least 80% (Miles and Huberman, 1994; Creswell, 2014; O'Connor and Joffe, 2020). Though our agreement falls slightly below the recommended 80%, we recognize that our coding scheme was complex as it involved both a substantial number of subcodes and simultaneous coding. The lower percentage of agreement is expected as prior literature suggests that, while allowing greater specificity in codes and multiple codes per excerpt is often necessary to capture meaningful representations of data, it regularly lowers intercoder reliability (Hruschka et al., 2004; Roberts et al., 2019; O'Connor and Joffe, 2020). However, our agreement of 74.48% indicates cohesion between reviewers.

## Results

The current study explored the role of parental socialization on Black adolescent girls' identity development. There were two main research questions: (1) How are Black girls making meaning of Black girlhood and in what ways might their identity processing be related to parental socialization? and (2) Are there differences in the identity processing and the content of reported parental socialization of Black girls in middle school compared to Black girls in high school?

**Research Question 1:** How are Black girls making meaning of Black girlhood and in what ways might their identity processing be related to parental socialization?

For the first research question, four themes emerged: (1) “Not like the other girls,” (2) Positive identities despite negative portrayals, (3) Awareness of stereotypes and their influence in K-12 Education, and (4) Affirmations in the face of anti-Blackness. Example quotes for each emergent theme and the subcodes associated with each quote can be found below in Table 2.

**Table 2:** Theme, example quotes, and subcodes for research question 1.

**Theme**	**Example quotes**	**Subcodes**
Not Like the *Other* Girls	“I mean, being a girl, I feel like when it comes to Black people, it's broken down into categories, in my opinion. …you have your Black males and your Black females”	• G-centrality • GRI-centrality
Positive Identities Despite Negative Portrayals	“Um what it means to be a Black girl, I feel like it means they have some sort of excellence. And we also have beauty and I feel like we are very underrated and taken advantage of.”	• GRI-private regard • GRI-public regard
Awareness of Stereotypes and Their Influence in K-12 Education	“Yes, Black girls are rowdier, ratchet, or they're always talking too much. Or they're always too loud or always trying to do everything and they should sit down.”	• GRI-public regard
Affirmations in the Face of Anti-Blackness	“I usually you know talk about it with my mom and certain things, but you know that was more when I'm younger, now that I'm older I kind of like tend to ignore it, or just you know, be like, I know that I look beautiful with my hair out and you have your hair out, and I can have my hair exact same way, so I just kind of like more brush it off and just like if that's what you feel then just don't look at me then.”	• GRS- pride and empowerment • GRS-hardship • GRI-assimilation • AS-school conflict

*G, Gender; GRI, Gendered Racial Identity; AS, Academic Socialization; GRS, Gendered Racial Socialization*.

### Not Like the *Other* Girls

Participants in this investigation emphasized their unique social positioning, being both Black and female. In fact, several girls expressed not being able to provide an answer for what being a girl meant to them generally as they only considered their experience as a Black girl, feeling that they did not share a collective experience with girls of all races.

“*I feel like it's very different from being like, a white girl, or you know, any other race or ethnicity. Um, I think that Black girls go through a lot more than any, like, than any other girl of a different race. I don't know. I just feel like we've been put down in a different way. I don't know how to explain it yeah. I think it's just different. It's very different.”*

    *Marsha, 17 years old, 12th Grade*

In participants' awareness of their unique social positioning, they often underscored how their identities led to experiences of marginalization or expectations that they would experience discrimination, inequity, and/or exclusion in the future due to being a Black woman. These were statements that girls expressed as a matter of fact, confident that these trials would come but also confident in their capacity to thrive anyhow.

### Positive Identities Despite Negative Portrayals

Despite awareness of hardships related to their group membership, participants also shared positive perceptions when asked what being a Black girl meant to them. Girls often described feeling a sense of pride in being a Black girl, highlighted their beauty, and emphasized the strength of Black girls and women.

“*I feel like being a Black girl means strongness, bravery… Strongness, bravery, smartness, everything. You're pushing through boundaries all the time. You're continuing to try to do the best that you can in order to show that we're not what the stereotypes that they put on us. We're not just oh someone who doesn't care about school and stuff like that. You have to continue to show that we are human just like everyone else, and we actually are very, very strong and not weak or all the labels that they tried to put on Black girls.”*

    *Katherine, 16 years old, 11th Grade*

In this example, Katherine expands on why she feels Black girls need to be strong. Her understanding of Black girlhood suggests that strength is a prerequisite to be able to maneuver a world in which Black girls are constantly fighting to disprove the negative portrayals and notions about Black girls and what they are capable of accomplishing. When asked how being a Black girl made her feel, the participant reiterated that it made her feel strong knowing what she can and has accomplished despite the barriers experienced by Black girls. She then states with unwavering conviction that she was “just as good if not better than any other race, any other person of color, or any other person of not-color,” further underscoring how girls meaning making around being a Black girl shapes their self-perceptions and motivations.

### Awareness of Stereotypes and Their Influence in K-12 Education

Many participants across developmental stage spoke of the stereotypes they were aware of or had encountered that were specific to Black girls. More common stereotypes were related to vocality and loudness of Black girls, policing of their bodies or suggestions about promiscuity, and Black girls' intelligence or academic potential. Of note, the stereotypes that girls provided in this study were primarily of a negative connotation.

“*Honestly in high school, like they're loud, ghetto, ratchet, very uneducated sometimes. They don't know what they're doing”*

    *Margaret, 14 years old, 9th Grade*

“*Oh, they're ghetto. Oh, they don't really care about school. All they do you know is, all they do is have kids and stuff like that…saying that Black girls, Black girls can't get through school. Black girls can't graduate. Black girls can't manage their hair. Black girls don't know how to, don't know how to be professional.”*

    *Katherine, 16 years old, 11th Grade*

Beyond girls' general awareness of these stereotypes, participants were also able to make direct connections between the negative portrayals of Black girls through stereotypes and their own negative school experiences.

“*Being a Black girl has impacted… It's impacted my schooling by… Some teachers—I used to go to this all White school, right? And some teachers would give me more work or put me in different classes, just because they think I'm not as smart or they think less, or they would make their own stereotypes for me. And they would do what they thought was right for her (teacher).”*

    *Ruth, 13 years old, 8th Grade*

In the above example, Ruth shares of her experience in a predominately white school in which she felt teachers assumed the level of her academic abilities based on her status as a Black girl. She goes on to elaborate that she believed her teachers felt she came from the “hood” so she must be “illiterate.” Ruth believed stereotypes about her race directly prevented her from participating in gifted programming saying, “because I am academically intelligent, like gifted, and they would look past that because of the color of my skin.” Interestingly enough, she chose not to involve her parents as she felt this was a particular problem that she wanted to overcome on her own. This choice is important to note as parents' awareness of these experiences guide their ability to intervene.

### Affirmations in the Face of Anti-blackness

All 12 participants mentioned that their parents helped them to navigate classroom or school-based conflict. Parents' conflict navigation strategies were tailored for both teachers and peers. In many of these instances, participants expressed that the conflicts were based in broader anti-Black sentiments, often involving their physical appearance. Girls relied on their mothers to affirm them as they processed how to interpret what they were experiencing at school and what to do next. Earlier affirmations from their parents appeared to be a source of comfort and a useful foundation for Black girls as they navigated anti-Blackness in later years of adolescence.

“*Yes, I usually you know talk about it with my mom and certain things, but you know that was more when I'm younger. Now that I'm older, I kind of like tend to ignore it, or just you know, be like, I know that I look beautiful with my hair out and you have your hair out, and I can have my hair exact same way. So, I just kind of like more brush it off and just like if that's what you feel, then just don't look at me, then.”*

    *Katherine, 16 years old, 11th Grade*

In the above example, Katherine describes her response to a group of White girls at her school that made jokes about her natural hair. In this passage, her classmates referenced her afro saying things like “Dang your hair is like crazy,” “You need to do something with it,” and “Why don't just slick it back or something?” In other instances, she gives the example of classmates saying, “Oh, my goodness, did you get electrocuted?” She makes mention of earlier years when she would have talked over this type of experience with her mother. However, now that she is older, she expressed a sense of agency and empowerment, attributed to those prior discussions. Instead of feeling the need to conform to the Eurocentric beauty standards of her peers, Katherine articulated confidence in her own beauty.

“*Being a Black girl makes me feel empowered but also… Less than almost, in society's views. I feel like I have the—just because how I was raised, I feel like I have the entire world open to me, and that I can do anything that I put my mind to, and I really work to do, but I am definitely going to have to work harder and knowing that just makes me feel a little bit stumped, but I still can go for whatever I want to go for.”*

    *Ruth, 13 years old, 8th Grade*

The above example illustrates Black girls' awareness and processing of the juxtaposition between negative societal views of Black girls and strong positive gendered racial identities. Further, this quote suggests that there may be an emotional toll associated with this awareness. When asked what being a Black girl means to her she says, “it will land me in a very low situation, because not only am I an African American, I'm also a female which is like double.” However, we see that even with this understanding of Black girlhood, she still maintains some feeling of empowerment. This participant credited her parents for this perspective, specifically believing that she can accomplish anything through perseverance. Though the participant acknowledges that her circumstances may be more difficult than others, she articulates her position that there are no limits to her success. This underscores how parental socialization both prepares youth for these experiences but also affirms them in ways that shape girls' negotiation of their multiple identities.

**Research Question 2:** Are there differences in the identity processing and the content of reported parental socialization of Black girls in middle school compared to Black girls in high school?

Three main themes emerged in our analysis of research question 2: (1) “(Dis)engagement with Traditional Gender Roles,” (2) “A Culture of Overcoming:” (3) “Silence as a Survival Strategy.” Example quotes for each emergent theme and the subcodes associated with each quote can be found below in Table 3.

**Table 3:** Theme, example quotes, and subcodes for research question 2.

**Theme**	**Example quotes**	**Subcodes**
(Dis)engagement with traditional gender roles	“I like to indulge in my more feminine side, so that means that I'll like more girly things. Or I like wearing dresses more and I like light airy colors. And I want this nice little house where I can do all my little things. I just find that really desirable”	G-private regard
A culture of overcoming	“Like the main thing about being Black to me is the culture because we have, we have like music, food, and cooking and dancing and holidays. And other things like that. So, it's good that you have all those things that you can like celebrate about being Black.”	R-private regard
Silence as a survival strategy	“My mom tells me to not talk back to her, but to at least explain to her the situation. And as long as I get good– as long as I did well in her class there shouldn't be any problems.”	AS-school conflict

*G, Gender Identity; R, Racial Identity; AS, Academic Socialization*.

### (Dis)engagement With Traditional Gender Roles

While much of what participants described of their identities throughout the interviews were related to their racial or gendered racial identity, there was a difference between middle and high school girls' choice to engage or disengage with traditional gender roles. Girls in middle school appeared to hold more closely to traditional gender roles and notions of femininity. For instance, middle school girls made mention of physical appearance such as wearing dresses, being more “emotional” than boys, and having to be creative in order to help others. However, girls in high school distanced themselves from these ideals, emphasizing that they did not need to conform to or participate in traditional gendered norms. Additionally, girls in high school described hardship and expectations that came from being a female compared to middle school girls.

“*I mean that we all have different sides of us different gender sides of us and for me personally, I like to indulge in my more feminine side, so that means that, I'll like more girly things or I like wearing dresses more and I like light airy colors and I want this nice little house where I can do all my little things. I just find that really… desirable, should I say.”*

    *Ruth, 13 years old, 8th Grade*

When first asked what being a girl means to her, Ruth responded that it means that she chooses to be feminine and that she must indulge in more of a feminine side. In the above excerpt she expands on this response by defining what encompasses a feminine side (dress, colors, connection to the home). Her understanding of girlhood was overwhelmingly positive, and she looked forward to the opportunity to perform girlhood and femininity in the ways she had been socialized. This was different from how Black girls in high school discussed girlhood, who more often emphasized that gendered expectations are something one must resist and fight against.

“*I feel like being a girl is being set to higher standards, high standards “Oh, you have to be, you have to do this, you can't do this” it's you have to go on a straight trail, but I feel like you don't have to though. You're put as to that you have to go on this trail. This how you have to have life. This has—how you have to get married. This is what you have to do when you get married. But I feel like that's not what you really have to do. You have to, you have to do anything you can do, and I feel like you, you have to try to stand– look at yourself as better than what people put on you. The labels that people put on you. You have to be continuing to be strong, also.”*

    *Katherine, 16 years old, 11th Grade*

In the above example, Katherine describes being a girl as being set to a higher standard that required compliance and left no room for variation in one's life path. Of particular importance were marital expectations and assumptions about how one must act as a wife. Disagreeing with more traditional ideals, this participant asserts that girls should feel comfortable deviating from these ideals and instead defy gender labels.

### A Culture of Overcoming

Participants in this study had meaningful and complex understandings of Blackness and more specifically what being Black meant to them. Within their varied responses to these questions, we find that middle school girls often referenced culture (which included things such as food, music, and familial gatherings). To them, Blackness is community driven and celebratory.

“*So, being Black means to me like…like the main thing about being Black to me is the culture. Because we have, we have like music, food, and cooking and dancing and holidays and other things like that. So, it's good that you have all those things that you can like celebrate about being Black.”*

    *Shirley, 11 years old, 6th Grade*

In contrast, high school participants reflected more on the individual efforts they must make to disprove negative portrayals of the collective. Their responses emphasized the desire to overcome and in fact, an urgency to do so as they considered the historical context. For example, one participant said “Remember, as you go through life because of the fact that that's something that keeps on pushing us, because we want to do better, for what our ancestors went through.” While girls noted the disadvantages and challenges that arise from responding to this call to action, girls reflected positively on the individual and collective strength exhibited by Black people.

“*Being Black to me, I feel like it means you have to…how do I want to word this? You have to show up and show out because most people think less of us so when you get in wherever you're going, whether it be an interview or sport. And you show them like that you actually know what you're doing then they be surprised. Then they know that Black people aren't what the stereotype of Black people are.”*

    *Mae, 14 years old, 9th Grade*

In this excerpt, we see that Mae believes that Blackness requires one to meet or exceed expectations in professional and leisure settings. This quote suggests that, because of her awareness of societal views about Black people, there is an internal pressure to outperform, guiding their interactions and experiences in school and professional settings.

### Silence as a Survival Strategy

As previously mentioned, all participants in this investigation (*n* = 12) discussed the ways they have sought support from their parents after experiencing conflict in schools, which were commonly a result of racism and/or sexism. Black parents consistently advised their daughters to ignore or not engage with teachers and classmates in these instances as a strategy to protect their daughters regardless of whether they were in middle school or high school.

“*They just said um that's how people…just how being a Black are. You're gonna have to deal with that for the rest of your life. So, they're just like this. It's okay. Just deal with it.”*

    *Gladys, 15 years old, 9th Grade*

In the above excerpt, Gladys describes her parents' strategy for responding to racist remarks from her classmates. During the interview she recalls how in middle school she often experienced her peers asking questions such as “Do you love fried chicken and watermelon?” and making mentions of her hair. Here her parents are attempting to prepare her for the bias and discrimination that she will encounter throughout her lifetime because she is Black. By telling her to “just deal with it,” they are diffusing the situation, asking her to not escalate it further.

*Participant: My science teacher she's okay. She teaches us what we need to learn. It's just… it's just what's the time… it's like she says that I'm talking, but I'm not talking at all and it's the other people. And she can't tell if somebody's talking or not because the masks. And it gets on my last nerve*.

    *Interviewer: So, what do you do about that?*

    *Participant: I just have to roll with it because I don't want to get written up or get called home*.

    *Interviewer: Hmm… Have you ever talked to your parents about that?*

    *Participant: Yes, I've talked about that to my mom and my father*.

    *Interviewer: What kind of advice do they give you?*

    *Participant: My mom tells me to not talk back to her, but to at least explain the situation to her. And as long as I get good– as long as I did well in her class there shouldn't be any problems*.

    *Alexa, 11 years old, 6th Grade*

In the above example, Alexa recalls an experience in class in which she is consistently called out and verbally reprimanded for talking in class even though she was not talking. Though she knows she is being singled out unjustly, she acknowledged that, for Black girls, an act as small as talking during class can be escalated to more serious disciplinary action. This was also evident in her mother's advisement that her daughter should not argue with the teacher. Instead, she suggests that her daughter discuss this with the teacher at another time. In this example, staying silent means that her daughter stays in the classroom and continues receiving science instruction instead of being sent outside of class as a disciplinary response. Silence in this example is strategic.

## Discussion

Using semi-structured interviews, this investigation explored the role of parental socialization on Black girls' identity development. Collectively, this investigation highlights how Black girls process their multiple marginalized identities. Notably, this investigation emphasized the role that parents play in helping their children navigate school conflict. Two key themes emerged from our findings—(1) Black girls' identity development and meaning making of Black girlhood and (2) the protective role of parental socialization.

### Black Girls' Identity Development and Meaning Making of Black Girlhood

Participants in this investigation shared thoughtful reflections on what it means to be a Black girl while also demonstrating a complex understanding of how their multiple social identities shape their lived experiences and future. Black girls reported that their Blackness was a central part of their identity and discussions of race guided much of their discussions on who they are. Of note, there were qualitative differences in girls' responses to what being Black meant to them for girls in middle and high school. Girls in middle school emphasized cultural tradition (e.g., music, foods, and community), while high school girls often highlighted the disadvantages faced by the Black community and the need to prove negative stereotypes wrong. Research indicates that as Black youth transition into adolescence, they become increasingly aware of differential treatment due to their race and report personally experiencing discrimination (Seaton et al., 2008; Hope et al., 2015; English et al., 2020; Tynes et al., 2020). Also, previous literature has indicated that Black youth are not only aware of academic race stereotypes but begin to endorse stereotypes at increasing rates across adolescence (Burnett et al., 2020). Black girls in this sample were highly motivated to resist stereotypes. This finding is supported by previous studies (e.g., McGee and Martin, 2011), which indicated that Black students were often focused on proving stereotypes wrong and sought the acceptance of those who thought less than of Black students in STEM contexts. As students grew older, McGee and Martin (2011) found that their focus shifted from proving stereotypes wrong for the approval of others to stereotype management in which they are internally motivated to succeed for themselves. Stereotype management can be defined as “a tactical toolkit for asserting their academic excellence in the face of being stereotyped” (p. 1,363). Future studies should examine Black girls' understanding of stereotypes, particularly as they transition into middle and high school and how it may motivate their academic decision making.

Contrary to the salience of Blackness, gender identity alone was not as central to participants' sense of self as they cited the vast differences in the experiences of Black girls compared to girls of other races. Thomas et al. (2011) found that when asking young Black adolescent girls and young adult women about their racial and gender identity separately, they often responded in a way that emphasized the complexity of their gendered racial identity status. The present study found qualitative differences. High school participants thought critically about society's perceptions of girlhood and vehemently sought to counter gendered expectations of how to perform girlhood. In contrast, girls in middle school spoke about gendered expectations and norms (e.g., clothing, colors, and homemaking). This difference may be in part to the high school girls' own lived experience as they described more hardship as a result of being a girl compared to middle school girls in this sample. Further, reflecting developmentally and life stage informed identity processes outlined in PVEST (e.g., Spencer et al., 2019), high school girls reported an awareness of norms and perceptions as well as developed response strategies.

Relatedly, participants often discussed expectations that they would encounter hardships at the intersection of racism and sexism. They often connected these expectations for hardship to their knowledge of stereotypes and lower educational expectations for Black girls and women. When participants were asked to share what stereotypes they believed existed about Black girls, they expressed that people described Black girls as being “loud, ghetto, extra, rowdy, uneducated, and unable to graduate.” The stereotypes that participants named are aligned with the decades of scholarship that highlighted the negative stereotypes of Black girls often held by teachers and school support staff (Morris, 2007; Archer-Banks and Behar-Horenstein, 2012; Neal-Jackson, 2018; Annamma et al., 2019; Carter Andrews et al., 2019; Gadson and Lewis, 2021).

Despite the awareness of these negative portrayals, girls in this sample overwhelmingly had positive feelings about being a Black girl and expressed what this means from an identity perspective. PVEST provides a lens to interpret these findings, emphasizing the need to understand the varied ways that youth may respond positively, even in the face of inequality (Spencer, 2006). Participants were proud to be a Black girl and described Black girlhood in ways that highlighted their beauty (e.g., natural hair), emphasized their strengths, and celebrated their contributions to the culture. Recent work by Rogers and Butler-Barnes (2022) found that adolescent Black girls' perceptions of their hair were an important part of their identity development, highlighting that hair was a vehicle of empowerment and resistance to Eurocentric beauty standards (Rogers et al., 2021), much like the girls in the present investigation. In our sample, feelings of empowerment were coupled with their desire to overcome barriers associated with their multiple marginalized identities. This aligns with previous research suggesting that persistence and heightened motivation to succeed, particularly in spaces where they feel devalued and underestimated, are adaptive responses by Black girls (e.g., Thomas et al., 2011; Archer-Banks and Behar-Horenstein, 2012). Additional scholarship is needed to discern how Black girls' persistence in these unwelcoming spaces impacts their career aspirations, educational attainment, and wellbeing.

### The Protective Role of Parental Socialization

Participants in this investigation often sought affirmation and support from their parents after experiencing instances of racism and/or discrimination from peers or teachers. For instance, one participant shared experiences of race-related bullying from classmates and how difficult it was for her to understand why she was being bullied. This participant goes on to discuss how conversations with her mom helped her to see that her Blackness may mean that she will experience racism but also provided her with strategies for how to cope when she does experience it. This parenting strategy maps onto the *preparation for bias* dimension of ERS. ERS is a parenting approach that seeks to prepare children for the potential of experiencing racism while still helping to cultivate a positive and healthy racial identity. In the current investigation, girls shared that the conversations with their parents earlier in life had aided in their confidence such that now when they encounter mistreatment due to their race in schools, they are still able to maintain a positive self-image. For the older children, they noted feeling empowered to disengage and not internalize the negative words and imagery. Research has found that in practice, Black families often use a mixture of socialization dimensions in tandem in which they both prepare children for discriminatory experiences (preparation for bias) and affirm their identity (racial pride; Harris-Britt et al., 2007).

A common theme in the parental messaging received from our participants was that silence and disengagement was the best survival strategy, especially in schools, when dealing with discriminatory encounters as well as racist teachers and students. Girls voiced not wanting to be written up or receive a call home as a reason for not engaging. This is not surprising as statistics indicate that Black girls have the fastest growing suspension rates and have rates higher than 67% of boys, which directly impacts their educational trajectories (Morris and Perry, 2016; Annamma et al., 2019; Ibrahim et al., 2021). Black girls are 4.19 times more likely to be suspended, 3.99 times more likely to be expelled, and 3.66 times more likely to be arrested compared to White girls (Epstein et al., 2020). Thus, when parents are suggesting silence as a strategy for navigating conflict in schools, they are also protecting their Black daughters from decreased classroom instruction time which impedes their academic progress and the potential of escalation of conflict that leads to involvement in the criminal justice system. Our study finding is in contrast to Smith-Bynum et al. (2016) investigation in which scholars explored Black mothers' responses and expressed strategies regarding their child experiencing discrimination perpetrated by a teacher compared to a store clerk. In this particular study, researchers found that mothers responded with more statements about intervening and advocating for their child when regarding teacher discrimination compared to if their child experienced discrimination at a mall. Additionally, mothers of daughters were more likely to deliver messages about advocacy compared to mothers of sons. This difference in parenting responses and strategies exhibited in the Smith-Bynum et al. (2016) investigation compared to the current study highlights the need for continued study into the antecedents and consequences of these two approaches to socialization. Lastly, much of our understanding of ERS in Black families has been in relation to retroactive discussions, meaning that it was in direct response to an encounter their child has already had or a racialized event that is widely discussed in their community or in the news (e.g., police sanctioned violence; protest). Work by Anderson and Stevenson (2019) notes the ways these conversations often lack discussion of how best to help children regulate their emotions in the moment and provide explicit coping skills. This is particularly important as we consider future work that explores how best to help Black girls navigate the racial stress and trauma they experience in educational contexts.

## Strengths and Limitations

There were several strengths of this investigation. First, the current study extends previous literature by centering Black girls' voices in an in-depth exploration into Black girls' meaning making of their identity and how their parents support their healthy development. Using a developmental lens, the current study was able to appraise whether there are qualitative differences in identity processing between Black girls in middle school and Black girls in high school. Though there were many important strengths of this investigation, there were also a few limitations. First, participants' reports of parental socialization messages and behaviors, while informative, also are girls' *received* messaging and doesn't necessarily guarantee that it fully represented all that parents expressed. Instead, what girls reported is more so the messaging they have deemed important, interpreted, and now integrated into their toolkit to navigate their various environments. Future research should consider the use of dyadic data collection to examine consistency in messaging and differences in interpretation of parental socialization messaging. Also, except for one participant, girls in this investigation attended a school that was at least 25% Black and over 50% ethnic-racial minorities. Thus, their experiences may be quite different from Black girls who attend schools that are predominately white. Additional research should be done to explore how school racial composition may influence girls' meaning making of Black girlhood.

## Implications

The current study has implications for the growing body of literature that seeks to center the voices of Black girls as their experiences are too often overshadowed and ignored. By further contextualizing their identity development in the broader academic context, this study provides meaningful suggestions for ways to better engage Black girls and their families in schools. Schools should seek to find additional ways to include parents and aim to strengthen family-school-community partnerships.

## Data Availability Statement

The datasets presented in this article are not readily available because of the use of personal stories and narratives of minors. Requests to access the datasets should be directed to marketab@mailbox.sc.edu.

## Ethics Statement

The studies involving human participants were reviewed and approved by University of North Carolina at Chapel Hill Institutional Review Board. Written informed consent to participate in this study was provided by the participants' legal guardian/next of kin.

## Author Contributions

MB contributed to the conceptualization, data collection, analysis, writing, reviewing, and editing of the manuscript. MM contributed to the analysis, review, and editing of the manuscript. MG and SC contributed to the review and editing of the manuscript. All authors contributed to the article and approved the submitted version.

## Funding

This work was supported by a Ford Foundation Dissertation Fellowship and a Dashiell Dissertation Start-up award that was awarded to the lead author.

## Conflict of Interest

The authors declare that the research was conducted in the absence of any commercial or financial relationships that could be construed as a potential conflict of interest.

## Publisher's Note

All claims expressed in this article are solely those of the authors and do not necessarily represent those of their affiliated organizations, or those of the publisher, the editors and the reviewers. Any product that may be evaluated in this article, or claim that may be made by its manufacturer, is not guaranteed or endorsed by the publisher.
