# Supplementary material for: “When I Think of Black Girls, I Think of Opportunities”: Black Girls' Identity Development and the Protective Role of Parental Socialization in Educational Settings
Source: Front Psychol. 2022 Jul 25;13:933476. doi: 10.3389/fpsyg.2022.933476 (PMC9358241; doi:10.3389/fpsyg.2022.933476)
Supplement: Supplementary file 1 [file Data_Sheet_1.docx]

Supplementary Material

# Supplementary Interview Protocol

1. We are going to start with a brief 5-minute exercise. During this exercise I want you to tell me about yourself. Whatever you may want to share. You can tell me as little or as much as you like. What have you always wanted people to know about you? I will start a timer and I will let you know once we hit five minutes. If you don’t need all five minutes, that’s okay too! Tell me when you’re finished sharing and we can move on to the rest of the interview. Do you have any questions?

2) What does being Black mean to you?

1. What does being a girl mean to you?
2. What does being a Black girl mean to you?
3. How does being a Black girl make you feel?
4. In your experience, how do other people view Black girls?
5. Are there any words or phrases you think of when you hear someone mention Black girls?
6. Do you know what a stereotype is? (If yes: ask them to define it; If no: Stereotypes are beliefs people often have about certain groups. For example, one stereotype could be that all tall people play basketball.)
7. Do you know of any stereotypes for Black girls? Are there any things you hear people say about just Black girls? I would love to hear more about that.
8. Where did you learn the things you know about being a Black girl?
9. Who told you these things?
10. How does hearing these things make you feel?
11. Has being a Black girl impacted your school experiences in anyway?
12. What is your favorite subject?
13. What is your least favorite subject?
14. Are you currently taking a math or science class?
15. If so, which classes are you taking?
16. Which math and science classes did you take last year?
17. What have been your experiences with science and math?
18. Have you ever discussed science and/or math with your parents?
19. Are there any differences between what your mom says and what your dad says?
20. Have you participated in any activities with your parents or been encouraged to participate in activities related to math and science by your parents? For example: Science museum, math games
21. What kinds of conversations have you had with your teachers about math and science?
22. What goals do you have after you complete high school?
23. STEM is a short way of saying science, technology, engineering, and math. There are all kinds of STEM jobs. For example, you can be a scientist, a nurse, a video game coder, an engineer who makes machines work, or even a mathematician who finds the perfect equation to get us to space. There are many STEM options available! Could you ever see yourself wanting a STEM job when you grow up? Why or why not?
24. Do you think there is anything stopping Black girls from going into STEM?
25. How can we best support Black girls who want to go into STEM?
26. Is there anything else you want to say that maybe my questions missed?
